# Hyperspectral and Chlorophyll Fluorescence Imaging to Analyse the Impact of *Fusarium culmorum* on the Photosynthetic Integrity of Infected Wheat Ears

**DOI:** 10.3390/s110403765

**Published:** 2011-03-28

**Authors:** Elke Bauriegel, Antje Giebel, Werner B. Herppich

**Affiliations:** 1 Department of Engineering for Crop Production, Leibniz-Institute for Agricultural Engineering Potsdam-Bornim, D-14469 Potsdam, Germany; E-Mail: agiebel@atb-potsdam.de; 2 Department of Horticultural Engineering, Leibniz-Institute for Agricultural Engineering Potsdam-Bornim, D-14469 Potsdam, Germany; E-Mail: wherppich@atb-potsdam.de

**Keywords:** chlorophyll defect, fungal diseases, non-destructive, non-invasive sensor application, potential maximum photochemical efficiency of PSII (F_v_/F_m_), *Triticum aestivum* L. ‘Taifun’

## Abstract

Head blight on wheat, caused by *Fusarium* spp., is a serious problem for both farmers and food production due to the concomitant production of highly toxic mycotoxins in infected cereals. For selective mycotoxin analyses, information about the on-field status of infestation would be helpful. Early symptom detection directly on ears, together with the corresponding geographic position, would be important for selective harvesting. Hence, the capabilities of various digital imaging methods to detect head blight disease on winter wheat were tested. Time series of images of healthy and artificially *Fusarium*-infected ears were recorded with a laboratory hyperspectral imaging system (wavelength range: 400 nm to 1,000 nm). Disease-specific spectral signatures were evaluated with an imaging software. Applying the ‘Spectral Angle Mapper’ method, healthy and infected ear tissue could be clearly classified. Simultaneously, chlorophyll fluorescence imaging of healthy and infected ears, and visual rating of the severity of disease was performed. Between six and eleven days after artificial inoculation, photosynthetic efficiency of infected compared to healthy ears decreased. The severity of disease highly correlated with photosynthetic efficiency. Above an infection limit of 5% severity of disease, chlorophyll fluorescence imaging reliably recognised infected ears. With this technique, differentiation of the severity of disease was successful in steps of 10%. Depending on the quality of chosen regions of interests, hyperspectral imaging readily detects head blight 7 d after inoculation up to a severity of disease of 50%. After beginning of ripening, healthy and diseased ears were hardly distinguishable with the evaluated methods.

## 1. Introduction

Many *Fusarium* spp. may cause serious grain contamination with mycotoxins (trichothecenes), mainly deoxynivalenol (DON) and 3-acetyl DON (3-ADON), on wheat [1]. Up to now, true infection of ears can only be determined with time consuming and expensive pre- and postharvest laboratory tests (serological rapid tests, Fast-DON-ELISA-test, counting methods; [2]). Thus, the ability to identify infections in the field could prevent exploitation of contaminated grain lots, finally, reducing toxin-burden of food- and feedstuffs.

Head blight, like other fungal and viral infections, is characterised by a complete destruction of the cellular integrity of the impacted tissues leading to cell death and degradation of chlorophylls. Damage is mostly accompanied by a transient increase in transpiration, followed by tissue desiccation. The resulting variation of tissue temperature has been successfully detected by thermal imaging, at least under controlled conditions [3]. On the other hand, chlorophyll degradation by means of spectral analysis in the visible range [4] allows detection of infected plant parts [5,6]. Application of spectral indices such as Normalized Difference Vegetation Index (NDVI), which has been shown to rapidly indicate plant stress [7], may provide a good discrimination between healthy and infected plant parts [8]. Including the NIR range in spectral analyses may increase the efficiency of discrimination because this wavelength range additionally includes information about tissue water content [9,10].

Providing further knowledge about local centres of infections, imaging methods are especially suitable for rapid and non-invasive identification of the effective stage of plants disease in the field. In this context, spectral imaging was used to diagnose viral infections [11] and to identify spatially variable physiological processes of leaves [12]. Hyperspectral analyses have been applied to detect fungal-based grain crop diseases [13–15]. Furthermore, by the combination of both hyperspectral reflection measurements and chlorophyll fluorescence analyses (CFA) the discrimination of yellow rust on winter wheat could have been improved to an accuracy of 94–95% [5].

CFA does not only measure the externally visible effects of infection-induced chlorophyll breakdown; it also provides comprehensive insight into potential and actual photosynthetic activity [16,17]. Photosynthesis is often considerably affected by both biotic and abiotic stresses at very early stages [18,19]. In addition, CFA imaging has already been universally applied, for instance for evaluation of the vitality of plant tissues [20,21], diseases like leaf rust and powdery mildew on cereals [22] or the infection by the tobacco mosaic virus [23].

For the detection of head blight, both methods have been shown to have great potential [24,25]. In this study, both imaging methods were applied in parallel to comprehensively analyse the respective ability of early detection of head blight disease in winter wheat ears both under laboratory and field conditions. Additional aims were: (1) to determine the highest possible accuracy of the detection of *Fusarium* infection; (2) to characterise the exact timeframe for meaningful head blight detection and (3) to determine the effect of the level of infection on detection accuracy.

## 2. Experimental Section

### 2.1. Materials and Experimental Design

Sixteen wheat caryopses (*Triticum aestivum* L. ‘Taifun’) were sown in eight pots (0.18 m × 0.18 m). From these, four pots were used for controls and four for infected samples, respectively. After germination, plants were cultivated in a greenhouse. After the start of flowering, plants were inoculated with a water suspension of *Fusarium culmorum* spores at a concentration of 250,000 spores per mL on three successive days. To guarantee the spread of germs, plants were kept at 20 ± 2 °C, high humidity (70%) and an illumination period of 12 h (high-pressure sodium-vapour lamps, SON-T Plus 400 W, Philips GmbH, Hamburg, Germany). Measurements started immediately after inoculation.

Developmental stages of ears were always graded according to the BBCH scale [26], which empirically describes plant development from dry seeds (BBCH 00) until the harvest product (BBCH 99). In the laboratory, plant infection levels were visually rated three times a week. Using sample pictures of infected ears, percentage infection of blighted spikelets per ear was estimated following [27]. Estimation of severity of disease occurred in distinct steps of 1%, 2%, 3%, 5% of damage at low infection levels and in 10%-steps at higher severity. All measurements were performed on intact plants. As a unit of visual rating of head blight disease pattern, the “severity of disease” (sod) was defined. During the course of this study three independent experiments were performed to comprehensively analyse both disease development and disease recognition accuracy.

### 2.2. Chlorophyll Fluorescence Imaging

Chlorophyll fluorescence imaging was performed with a modular system (FluorCAM 700MF, PSI, Brno, Czech Republic) measuring sequences of fluorescence images with a user-defined timing of set points, measurement intervals and irradiance [21,28,29]. Basic fluorescence F_0_ was induced by two panels of super-bright orange light emitting diodes (λ_max_ = 620 nm, 345 LED per panel; approx. 3 μmol m^−2^ s^−1^). Maximum fluorescence (F_m_) was triggered by short-term (1 s) saturation light pulses (max. 2,500 μmol photons m^−2^ s^−1^) generated by an electronic shutter-equipped halogen lamp (250 W).

The ratio of variable fluorescence (F_v_ = F_m_ – F_0_) to maximum fluorescence, F_v_/F_m_, is an indicator of the potential maximum photochemical efficiency of photosystem II. It ranges between 0 (chlorophyll-less, dead plants) and 0.84 for healthy, intact plant parts under optimal conditions [17]. F_v_/F_m_ is well-known as a valuable tool to determine both capacity and stability of photosynthesis [29,30]. A CCD camera with a F1.2/2.8–6 mm objective and a filter system (high pass 695 nm, low pass 780 nm) recorded fluorescence images (12-bit, 512 × 512 pixel; maximal frequency 50 images s^−1^) synchronously with the weak, non-actinic measuring-light pulses. The system was controlled by the FluorCam software (PSI, Brno, Czech Republic). In the laboratory, measurements lasted 4 s (F_0_: 3 s duration, 4 frames recorded; F_m_: 1 s duration, 25 frames recorded) and were performed on plants, dark-adapted for 10 min. Six samples of both infected and control plants were continuously recorded in time series experiments. In addition, fifteen plants with pronounced head blight symptoms were investigated at BBCH stage 75. All plants were measured from the side at a distance of 0.2 m between ears and CCD-camera.

In addition, chlorophyll fluorescence imaging was applied on artificially infected winter wheat plants (BBCH stage 77/79) of the cultivars ‘Cardos’, ‘Winnetou’ and ‘Drifter’ (all resistance class 5) directly in the field. To eliminate the effects of direct sunlight on fluorescence and to pre-darken (10 min) the plants, they were partially shielded with a paper box (approx. 0.9 m × 0.9 m × 0.9 m) during measurements. The duration of the fluorescence measurements was reduced to 2 s (*i.e.*, 1 s for F_0_ measurements and 1 s for F_m_). In total, 50 ears of varying levels of infection were investigated. Only optimally illuminated images with clearly distinguishable ears (n = 30) were further analysed in this experiment.

For a further evaluation and derivation of severity of disease (sod), in the chlorophyll fluorescence images, all F_v_/F_m_-pixel values were allocated to “efficiency classes” of photosynthetic activity at steps of 0.05. In addition, they were accumulated to a “cumulative F_v_/F_m_” (%), starting from the lowest values (0.00).

### 2.3. Hyperspectral Imaging

The laboratory hyperspectral imaging device recorded reflection spectra in a wavelength range of 400 to 1,000 nm with a spectral resolution of 2.5 nm. General pixel-resolution of the camera was 1,392 × 1,024 px; however, two pixels per axis were combined to yield an effective resolution of 696 × 512 px. The field of view achieved generally resulted in a spatial resolution of approx. 0.4 mm per pixel. The system comprised a spectrograph (ImSpector V10E, Spectral Imaging Ltd., Oulu, Finland), a 12 bit, digitally temperature-compensated b/w camera (Pixelfly qe, PCO AG, Kelheim, Germany) and an accessory rotating mirror with a micro-step motor. The hyperspectral camera stored the spectra of all pixels of an image line by line. A program, developed under LabView 8.2 (National Instruments Corporation, Austin, TX, USA), was used to control the camera system and for data pre-processing, including the black/white calibration of the spectra. For this calibration, images for the black and the white adjustment were recorded with each measurement. The b/w-balance [(sample–black)/(white–black)] was performed for the entire frame during the following conversion of the hyperspectral images into the byte stream format. Heterogeneities in the pixel response across the sensor area were generally rather low and, therefore, not compensated for. The samples were illuminated with a stabilised halogen lamp (150 W). In addition to the six ears per variant used for chlorophyll fluorescence analysis, further six plants were examined by hyperspectral imaging with a time lag of approximately two days during three weeks (total n_infected_ = n_controls_ = 12). All plants were recorded from the side at a distance of 0.5 m between plant and camera. To avoid vibrations, the ears were fixed on a black background. Exposure time, adjusted for the respective samples, was in the range of 20 to 25 ms; a complete record lasted 20 to 30 min.

### 2.4. Data Analysis

The classification of diseased and healthy areas was performed with the software ENVI (Research Systems Inc., Boulder, CO, USA) by means of monitored classifications in the “Spectral Angle Mapper” (SAM) evaluation algorithm. SAM compares the classifying spectrum of an image with a reference spectrum. The classes are allocated according to their similarity. The distinction of two reflection spectra is described with an angle, which span between related vectors [31,32]. In this paper, a vector in a multidimensional space (512 bands) was used. In addition, SAM was chosen because it is insensitive to variations of illumination [33].

The threshold of similarity of compared spectral angles was 0.1. For this purpose, regions of interest (ROIs) were established as the bases for the classification according to the two classes, diseased (8 ROIs) and healthy (10 ROIs). In a three-band false colour image (450 nm, 550 nm, 650 nm), diseased and healthy areas could be distinguished by visual inspection, which facilitated the proper manual setting of ROIs. These 18 ROIs was used to build an endmember, applied in the further calculations. To consider the effect of plant development, single hyperspectral images were repeatedly used as training images for setting ROIs. These images were excluded from later evaluation. After classification, the relative portions of pixels per image belonging to the healthy, diseased and unclassified object classes were determined. In this context, all pixels which could not be allocated to the defined classes “healthy” and “diseased” were assigned to the class “unclassified“. The proportion of unclassified pixels was calculated as the difference between 100% and the sum of healthy and diseased pixels.

## 3. Results

The development of *Fusarium* infection, rated as severity of disease (sod), and the relevant BBCH stage of plants at respective days after inoculation (dai) are shown for the first time-series experiment in Table 1. The first symptoms of the disease became visible at the BBCH stage 71/75 (7 dai), first ripening symptoms developed in the BBCH stage 81.

### 3.1. Chlorophyll Fluorescence Imaging

**Figure 1.**
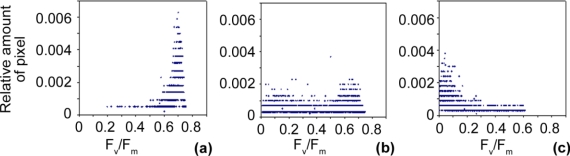
Pixelwise distribution of the maximum photochemical efficiency (F_v_/F_m_) of **(a)** weakly (2%), **(b)** medium (50%) and **(c)** strongly infected (90%) wheat ears.

Distribution analyses of chlorophyll fluorescence images of ears with a sod between 2 and 100% revealed that, in weakly diseased ears (2%), as in healthy ears, pixel-values of photosynthetic efficiency (F_v_/F_m_) concentrated in classes of high efficiency [0.55–0.75; Figure 1(a)]. The pixelwise F_v_/F_m_-distribution was nearly identical in control plants and very weakly diseased ears (data not shown).

In medium infected ears (50%), the distribution of F_v_/F_m_ broadened [Figure 1(b)] due to the co-existence of both healthy (pixel-value range 0.40–0.75) and diseased tissues (pixel-value range 0.00–0.40). In strongly infected ears [Figure 1(c)], pixel-values of photosynthetic efficiency only concentrated in the low-value range (0.00–0.20).

**Figure 2.**
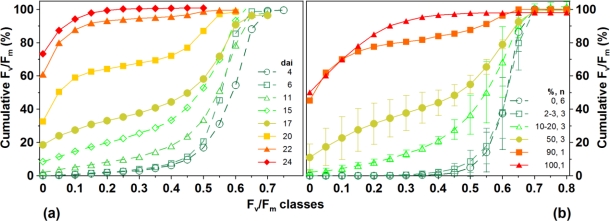
Cumulative F_v_/F_m_-values (%) **(a)** during several stages of head blight development of a single representative ear **(b)** Average cumulative percentage of F_v_/F_m_-values of ears at different levels of infection at dai 11.

Cumulative F_v_/F_m_ appropriately characterised the head blight development of representative ears (Figure 2). At an early infection state [Figure 2(a), dai 4–6], only a few pixel values were found in low efficiency classes, while 80% of F_v_/F_m_-pixel values concentrated in high photosynthesis efficiency classes (>0.6) in a healthy or weakly diseased (2–3% infection) ear. Continuous development of infection during the course of the experiment from dai 6 to dai 22 could be easily identified by an increasing concentration of accumulated F_v_/F_m_–values in low efficiency classes. This means that moderately diseased ears (40–60%) comprised both photosynthetically active and inactive areas. In contrast, cumulative F_v_/F_m_ obtained only low efficiency classes (<0.3) if the ear was strongly diseased (90%) 22 d after infection. This is also verified if the variation of average cumulative F_v_/F_m_ values of various plants of different sod is analysed [Figure 2(b)]. In this context, a cumulative F_v_/F_m_ at 0.3 seems to represent a relevant threshold to differentiate diseased and healthy ears.

**Figure 3.**
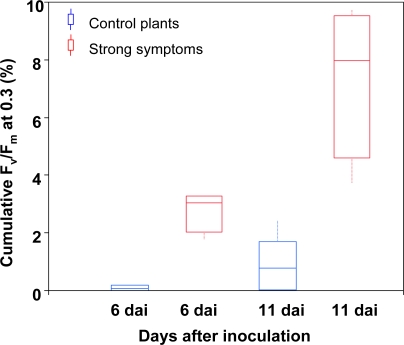
Cumulative F_v_/F_m_ values at 0.3 of controls (blue) and strongly (defined as 5% sod at dai 11) infected ears (red).

During early infection and at low infection state (sod 2% to 3%), cumulative photosynthetic efficiency of investigated ears overlapped indicating that visual rating and CFA imaging did not obtain completely identical results [Figure 2(b)]. On the other hand, controls and clearly infected ears (sod *ca.* 5% at dai 11) could be successfully differentiated by analysing the cumulative F_v_/F_m_ classes at 0.3 (Figure 3). Even at the first day of measurement (dai 6) the cumulative proportion of low efficiency classes was 3% higher than the control value, and rose to a median of nearly 8% within one week (dai 11). Up to a sod of 4%, F_v_/F_m_ of diseased and control ears did not differ. Even with a sod of 10%, plants showed only minor visible symptoms of head blight one week after inoculation.

**Figure 4.**
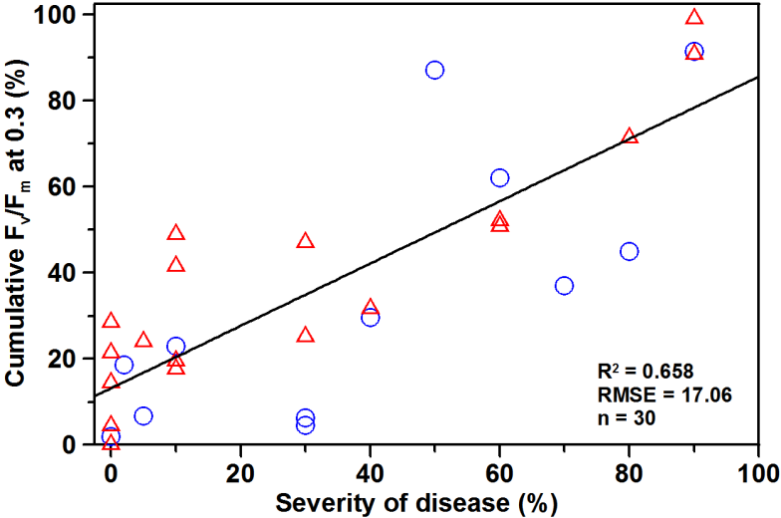
Correlation between the cumulative F_v_/F_m_ at 0.3 and the severity of disease obtained from visual rating (circles: 23 June 2009, triangles: 24 June 2009).

Field application of CFA imaging yielded in more variable differentiation of ears according to the severity of disease than laboratory studies (Figure 4). Discrepancy between visual inspection and CFA was more pronounced at low infection (sod < 30%). Nevertheless, ears with medium (40–50%) and high (70–80% and 90%, respectively) sod could easily be identified. Overall, correlation between the cumulative F_v_/F_m_ and sod was high in this experiment yielding a coefficient of determination of 0.658 (Figure 4).

### 3.2. Hyperspectral Measurements

**Figure 5.**
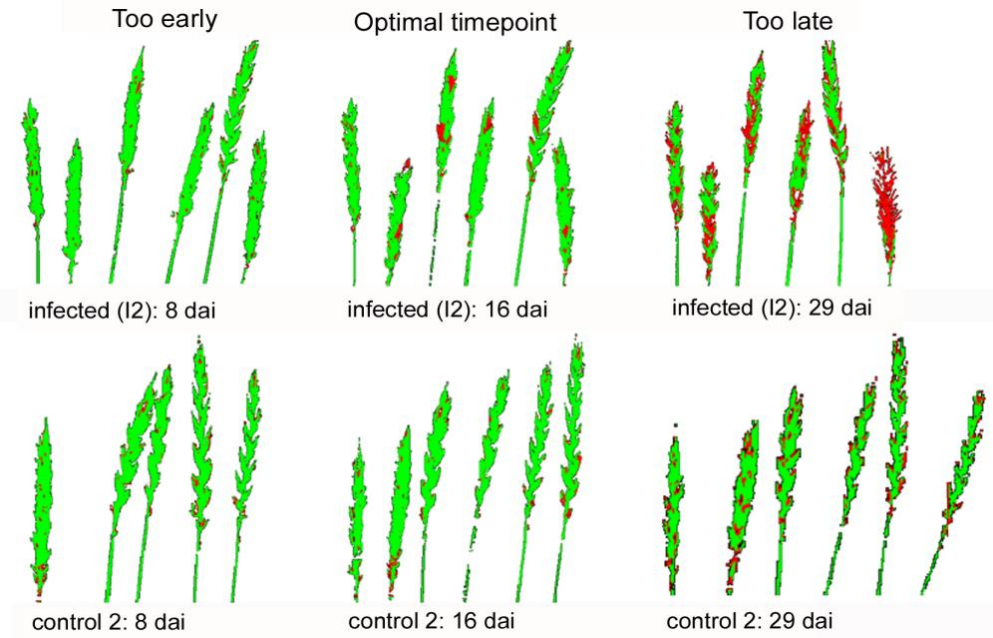
Samples of classification results using SAM classification (green: healthy classified tissues, red: diseased classified tissues). Upper row: infected ears, lower row: controls).

Time series experiments showed that differentiation by hyperspectral imaging was most effective at 14 ± 2 dai (Figure 5, dai 16 shown). It was less effective and, hence, results less reliable soon after infection and, again, after the beginning of maturation *ca.* four weeks after infection.

**Figure 6.**
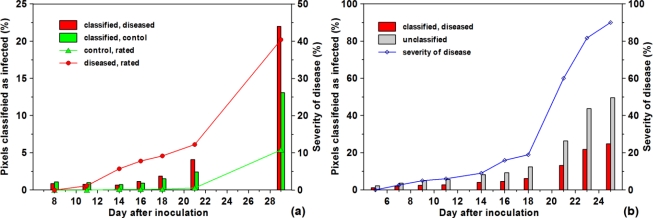
**(a)** Results SAM-based classification of infected and control ears (n = 12) in comparison with the severity of disease obtained by visual rating during the course of infection development. **(b)** Proportion of pixels classified as infected and those unclassified by the SAM algorithm in comparison with the severity of disease obtained by visual rating (n = 6). Results of healthy ears are not shown.

Results of classification of the levels of infection, obtained from hyperspectral imaging by the SAM algorithm, reflected those of the visual rating [Figure 6(a)].

For this comparison, proportions of the whole ears, classified as diseased, were related to the total number of classified pixels (healthy + diseased, see Figure 5). During early infection, starting from BBCH-stage 75, the sod obtained by spectral classification was always lower than that rated visually; it became closer at the beginning of ripening. Head blight was well separated from healthy tissues after the onset of ripening (from BBCH 81, dai 21). However, at this stage, pixels, which previously were classified as healthy, were now increasingly ascribed as unclassified [Figure 6(b)]. Generally, results of the SAM evaluation algorithm applied to hyperspectral image analysis were highly correlated (R^2^ = 0.964) with visually evaluated sod (data not shown). In all cases, the quality of classification strongly depended on the appropriate setting of ROIs. For this purpose, it is necessary to inspect the respective region of the image at the highest resolution.

### 3.3. Effects of Ear Development on Quality of Head-Blight Detection

**Figure 7.**
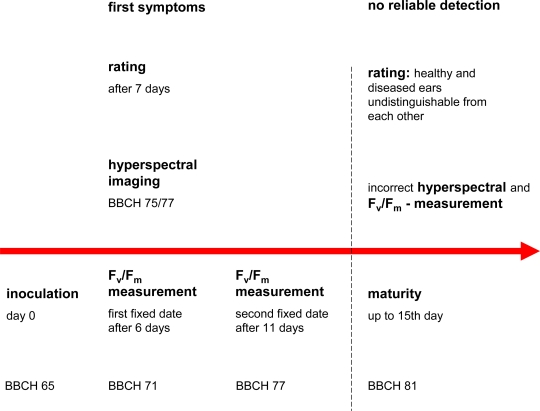
Time bar for the detection of head blight under indoor conditions.

The presented results indicate that during early ear development (starting from BBCH-stage 75), initial symptoms of infection can be eye-detected at 7 dai (Figure 7).

Supervised classification of hyperspectral images in visual range identified first head blight symptoms at the same time. Most efficient classification of *Fusarium*-affected ears was possible in the BBCH-stage 75 to 77. With progressing maturation, the number of ears that could not be classified correctly largely increased. However, these limitations were also partially valid for CFA imaging. On the other hand, F_v_/F_m_-measurements detect symptoms of infection of ears earlier than visual rating and hyperspectral imaging. In general, highest accuracy of detection of *Fusarium* infection may be achieved if two successive measurement dates were performed at the growth stage “medium milk” (grain content is milky, BBCH 71–77; dai 6 to 11).

## 4. Discussion

To the best of our knowledge, this is the first investigation on the combined application of chlorophyll fluorescence and hyperspectral imaging for the early *in vivo* detection of head blight disease in winter wheat. It could be convincingly shown that both methods can indentify *Fusarium* infection of wheat ears non-invasively and with high reliability at a very early stage of disease.

Based on the physiology of photosynthesis, chlorophyll fluorescence imaging allowed detection of the initial phase of tissue damage. After the penetration of kernels by the mycelia, there are distinct cellular changes such as degeneration of cytoplasm and cell organelles, decomposition of the host’s cell walls and deposition of material in vessel walls of the diseased ears [34]. Infection may lead to a complete inhibition of the metabolic activity, including a pronounced disturbance of photosynthetic performance. This can be easily identified by a rapid decline in the photochemical efficiency in the infected ears [24], even before visible chlorophyll degradation occurs.

Chlorophyll fluorescence imaging is a well-established effective tool to comprehensively assess the development and the effects of bacterial, fungal and viral infections on leaves of many crop plants (e.g., [8,23,35]). One topic of this study was to optimize both application of this technique and analysis of obtained results for rapid and early detection of head blight on wheat ears in laboratory and in field. To establish the level of infection of intact ears, the potential maximum photochemical efficiency of PSII (F_v_/F_m_) was applied. In contrast to the analysis of absolute F_v_/F_m_ values, the statistical evaluation of its relative distribution in the entire image provided a successful approach for this purpose. The broadening of the overall distribution of F_v_/F_m_ with developing infection closely reflected the increased number of diseased kernels per ear.

The analysis of the cumulative F_v_/F_m_ allows an accurate evaluation of the changed distribution pattern. Considering a cumulative percentage at 0.3 as the differentiation threshold, levels of infection can be differentiated in 10%-steps. Hence, even from the sixth dai, infected and control plants could be effectively separated in laboratory experiments.

Using this approach, it could be shown that the fungi seriously affect photosynthetic performance and, thus, chlorophyll fluorescence at an early stage. This has also been reported by [8,36] for leaf pathogens. For instance, [8] found reduced F_v_/F_0_-values two to three days before leaf rust and powdery mildew infection became visible on leaves of winter wheat.

In several studies, other image analysis approaches have been applied. Investigating yellow rust infection on wheat, [5] recorded fluorescence images at 550 and 690 nm. From the relative signal intensities at these two wavelengths, the authors built a disease index (f_G_) and defined pixels exceeding the f_G_ value of 0.65 as “diseased”. For each leaf of Tulip Breaking Virus (TBV)-infected plants, [37] calculated mean and standard deviation in photochemical efficiency classes (0–1) of fluorescence images. With this procedure, they got higher error rates (31–46%) than found by [5] or obtained in the present study. Classifying infected or healthy ears by the cumulative F_v_/F_m_ at 0.3, as used in this study, is the fastest method of analysis.

The current techniques of chlorophyll fluorescence imaging for identification of head blight in the field certainly need improvement. Due to their complex physiological nature [16], the fluorescence signals directly depend on the prevailing photosynthetic photon flux density. Hence, fluctuating light and direct exposure to sunlight must be avoided during measurements. Furthermore, before measurement of F_0_ and F_m_, plants need to be dark-adapted [5,17]. As shown in this study, these requirements can certainly be achieved.

Although the applied measuring system was developed for use in the laboratory, a suitable (R^2^ = 0.658) correlation between fluorescence analysis and visible head blight inspection has been obtained under field conditions. The reduction of correlation quality may be due to the high subjectivity of visual rating. The scale applied for visual rating had a step size of 10%; therefore, the absolute rating error would be 10% in the worst case. Methodological problems with the FluorCam measurements could not be entirely excluded but can be largely minimised by proper handling of the system.

Furthermore, movement of ears, induced by strong wind during recording of the sequences of F_0_ and Fm images, may result in non-overlaying frames of these two parameters. Therefore, overall recording time was reduced to 2 s. Nevertheless, peripheral areas of ears, which were influenced by wind, may have incorrectly low F_v_/F_m_. However, the resulting poorly observable marginal regions at the border area of the ROIs may be excluded from further data analysis. Also, incomplete, uneven shading in the measuring box occasionally provide another problem, leading to an overestimated basic fluorescence and, hence, erroneously low F_v_.

Elimination of all identified outliers reduced the amount of analysable ears by one third. As a consequence, the degree of determination of the correlation between fluorescence analysis and visible disease inspection rose to R^2^ = 0.80. This clearly indicates the high potential of chlorophyll fluorescence imaging for non-invasive disease detection after further improvement of measuring technique and protocols.

In case of hyperspectral imaging, the SAM classification algorithm used resulted in good and reliable detection of diseased ears. According to [32] SAM has a great potential for analysis in multi- and hyperspectral imaging. Nevertheless, the best classification method always depends on the complexity of the initial sets of data. To distinguish between *Fusarium*-infected and non-infected wheat ears, [38] evaluated RGB images with only three available channels and achieved better classification results by applying the Maximum-Likelihood-Method compared to the application of SAM. With increasing spectral information, other classification methods such as SAM [39], k-Nearest Neighbour, Decision Tree or Support Vector Machines [40] are certainly indispensable. However, basically improved imaging techniques, which allow reproducible and reliable data recording, may represent a necessary first step to optimize and, most important, automatize disease detection.

In general, hyperspectral images have a much higher information density than RGB. Hyperspectral image analysis is based on the entire spectral range investigated and it not only refers to three colour channels. Using spectral images (400–750 nm), [41] clearly separated different ripening stages of tomatoes, whereas application of RGB-images was not successful. In addition, the use of distinct ratios of different wavelengths for disease control and quality analysis has been widely reported [42–44]. In this context, to apply spectral imaging under field conditions, data gained by hyperspectral systems may be used to extract relevant wavelength ranges for rapid multispectral devices.

An important problem with the disease classification by spectral imaging is the choice of the correct stage of development; otherwise the results may be inaccurate. If the measurements start too early, floral residues (anthers) and sterile ears caused by growth disorders are classified as diseased. Hence, in initial phases of the present studies, the low level of infection (*ca.* 3%) classified by SAM on healthy ears (see Figure 6) was not based on head blight but reflected developmental disorders such as barren middle ears or tips. This means that damage other than that caused by *Fusarium*, were inevitably classified as diseased. Both types of damages could not be readily distinguished.

Classification results could, in some cases, be improved by either choosing different angle’s radian specific to the respective object classes or by manually adjusting the angle’s radian to lower values, e.g., to 0.05. However, such variations showed to be advantageous only for ears in the BBCH stages 89, because it also decreased the total number of classified pixel.

With the incipient ripeness, the spectra of healthy and diseased ears become more similar, which, again, leads to an increasing misclassification. Unclassified pixels clearly reflect the progressing degradation of chlorophyll during maturation, which occurred in healthy ears without the distinct spectral signature of infected kernels. In the classification procedure applied, such a class has not been specified but will be a next step of optimization. This has to be verified on the control plants which were free of a *Fusarium* infection.

Both methods investigated here are suitable for the detection of head blight. However, the next step to improve the accuracy of classification should be the dynamic combination of both methods and the addition of form and spot parameters, as proposed by [37]. A highly accurate classification is very important, because minimal levels of infection can lead to a contamination of major harvest lots with the poisonous mycotoxins of the *Fusarium*-fungi.

## 5. Conclusions

Laboratory as well as in-field measurements were performed to investigate the applicability of chlorophyll fluorescence and hyperspectral imaging for the detection of head blight. Under laboratory conditions, chlorophyll fluorescence imaging detects even very low levels of infection (*ca.* 5%) as early as 6 dai; visual classification is only possible beginning from 7 dai.

One single measurement enables a distinction between infected and healthy ears, provided the disease is sufficiently strong. However, two measurement dates are recommended to reliably detect even a minimal infestation and to eliminate possible errors of measurement. By the use of the cumulative Fv/Fm threshold of 0.3, the severities of infection can be detected with an accuracy of 10% under laboratory conditions. Under field conditions a differentiation between low (0–10%), medium (40–50%) and high (70–80% and 90%, respectively) level of infection can also be described with a linear model (R^2^ = 0.658, RMSE = 17%). Yet, the accuracy may rise up to 80% after data pre-processing including the elimination of outliers.

The application of the SAM evaluation algorithm yielded relatively good classification results. Nevertheless, the number of unclassified pixels increased during ear development.

The correct growth stage for spectral measurements and classification is therefore very important. From the BBCH-stage 81 (beginning of ripening) on, a distinction between healthy and diseased ears by the methods discussed above is limited.

## Figures and Tables

**Table 1.** Representative example of plant development (BBCH stage) and rated disease symptoms of head blight (severity of disease, sod, n = 6).

**dai**	**5**	**7**	**9**	**11**	**14**	**16**	**18**	**21**	**23**	**25**
BBCH	65/71	71/75	75	75/77	77/79	79/81	79/81	81/85	85	89
sod (%)	0	3	5	6	9	16	19	60	82	90
